# Telomere and Centromere Staining Followed by M-FISH Improves Diagnosis of Chromosomal Instability and Its Clinical Utility

**DOI:** 10.3390/genes11050475

**Published:** 2020-04-27

**Authors:** Radhia M’kacher, Bruno Colicchio, Claire Borie, Steffen Junker, Valentine Marquet, Leonhard Heidingsfelder, Kevin Soehnlen, Wala Najar, William M. Hempel, Noufissa Oudrhiri, Nadège Wilhelm-Murer, Marguerite Miguet, Micheline Arnoux, Catherine Ferrapie, Wendy Kerbrat, Andreas Plesch, Alain Dieterlen, Theodore Girinsky, Philippe Voisin, Georges Deschenes, Anne-Claude Tabet, Catherine Yardin, Annelise Bennaceur-Griscelli, Michael Fenech, Patrice Carde, Eric Jeandidier

**Affiliations:** 1 Cell Environment, DNA Damage R&D, 75020 Paris, France; kvsoehnlen72@gmail.com (K.S.); wala.najar@cell-environment.com (W.N.); williamhempel824@gmail.com (W.M.H.); philvoisin@free.fr (P.V.); 2 IRIMAS, Institut de Recherche en Informatique, Mathématiques, Automatique et Signal, Université de Haute-Alsace, 68093 Mulhouse, France; bruno.colicchio@uha.fr (B.C.); alain.dieterlen@uha.fr (A.D.); 3 APHP-Service D’hématologie Oncohématologie Moléculaire et Cytogénétique Hôpital Paul Brousse Université Paris Saclay/ Inserm UMR 935, 94800 Villejuif, France; claire.borie@aphp.fr (C.B.); noufissa.oudrhiri@aphp.fr (N.O.); micheline.arnoux@aphp.fr (M.A.); catherine.ferrapie@aphp.fr (C.F.); wendy.kerbrat@aphp.fr (W.K.); annelise.bennaceur@aphp.fr (A.B.-G.); 4 Institute of Biomedicine, University of Aarhus, DK-8000 Aarhus, Denmark; sjunker@biomed.au.dk; 5 Service de Cytogénétique, Génétique Médicale, et Biologie de la Reproduction Hôpital de la Mère et de l’Enfant, CHU Dupuytren, 87042 Limoges, France; valentine.marquet@chu-limoges.fr (V.M.); catherine.yardin@unilim.fr (C.Y.); 6 MetaSystems GmbH, Robert-Bosch-Str., 6 D-68804 Altlussheim, Germany; lheidingsfelder@metasystems.de (L.H.); aplesch@metasystems.de (A.P.); 7 Faculté de Médicine, Université Paris Descartes, 75005 Paris, France; 8 Service de Génétique Groupe Hospitalier de la Région de Mulhouse et Sud Alsace Mulhouse, 68070 Mulhouse, France; nadege.murer@ghrmsa.fr (N.W.-M.); mmiguet@gmail.com (M.M.); jeandidiere@ghrmsa.fr (E.J.); 9 Department of Radiation Oncology, Gustave Roussy Cancer Campus, 94800 Villejuif, France; theogirinsky@me.com; 10 Nephrology Department, APHP-Hopital Robert Debré, 75019 Paris, France; georges.deschenes@aphp.fr; 11 Cytogenetic Laboratory, APHP-Hopital Robert Debré, 75019 Paris, France; anne-claude.tabet@aphp.fr; 12 School of Pharmacy and Medical Sciences, University of South Australia, Adelaide, SA 5000, Australia; mf.ghf@outlook.com; 13 Genome Health Foundation, North Brighton, SA 5048, Australia; 14 Department of Hematology, Gustave Roussy Cancer Campus, 94800 Villejuif, France; dr.pcarde@gmail.com

**Keywords:** telomere, centromere, dicentric chromosome, chromosomal instability, cancer, genetic syndrome

## Abstract

Dicentric chromosomes are a relevant marker of chromosomal instability. Their appearance is associated with telomere dysfunction, leading to cancer progression and a poor clinical outcome. Here, we present Telomere and Centromere staining followed by M-FISH (TC+M-FISH) for improved detection of telomere dysfunction and the identification of dicentric chromosomes in cancer patients and various genetic syndromes. Significant telomere length shortening and significantly higher frequencies of telomere loss and deletion were found in the peripheral lymphocytes of patients with cancer and genetic syndromes relative to similar age-matched healthy donors. We assessed our technique against conventional cytogenetics for the detection of dicentric chromosomes by subjecting metaphase preparations to both approaches. We identified dicentric chromosomes in 28/50 cancer patients and 21/44 genetic syndrome patients using our approach, but only 7/50 and 12/44, respectively, using standard cytogenetics. We ascribe this discrepancy to the identification of the unique configuration of dicentric chromosomes. We observed significantly higher frequencies of telomere loss and deletion in patients with dicentric chromosomes (*p* < 10^−4^). TC+M-FISH analysis is superior to classical cytogenetics for the detection of chromosomal instability. Our approach is a relatively simple but useful tool for documenting telomere dysfunction and chromosomal instability with the potential to become a standard additional diagnostic tool in medical genetics and the clinic.

## 1. Introduction

Chromosomal instability is defined as a process of the progressive accumulation of numerical and structural chromosomal aberrations via chromosome segregation error during cell division. Chromosomal instability drives cancer initiation and evolution [1,2]. Chromosomal instability has proven to be an essential biomarker for patients with cancer, inflammatory diseases, and individuals in otherwise healthy populations exposed to genotoxic agents, as well as their progeny [3,4]. Ample evidence acquired during the past decades has demonstrated the predictive and prognostic value of chromosomal instability as a biomarker for treatment response and clinical outcomes of these populations [5,6,7,8,9,10].

Substantial progress has been achieved in the detection and identification of chromosomal instability. Conventional karyotyping involving, e.g., Giemsa banding (G and R-banding) or inverted DAPI (4′,6′-diamidino-2-phenylindole) staining is still the most precise and reliable technique used in research and clinical settings [11]. Despite its limitations in the detection of aberrations, i.e., less than one megabase, as well as karyotype heterogeneity, conventional karyotyping has proven to be an important tool for clinical diagnosis, especially among onco-hematology patients [12,13,14] and those with genetic disorders [15]. The introduction of fluorescence in situ hybridization (FISH) in cytogenetic analyses has immensely improved the detection of numerical and structural aberrations in metaphases, as well as interphases [16]. However, genomic analysis (NGS or micro-arrays) has lately made a sensational entrance into the clinical field [17]. Nevertheless, these approaches have their shortcomings, in that they fail to detect the level of karyotype heterogeneity, as well as chromosomal instability involving repeated sequences, telomeres, and centromeres [18]. 

Among the three main pathways of chromosomal instability, i.e., random breakage, telomere fusion, and centromere fission, the last two are generally underestimated using standard cytogenetic techniques [19]. 

Telomeres protect the ends of chromosomes, securing genome stability and integrity. Dysfunction of the telomere nucleoprotein complex can expose free chromosome ends to the DNA double-strand break (DSB) repair machinery, leading to telomere fusion and dicentric chromosome formation. Thus, the consequences of telomere loss or dysfunction can promote chromosomal instability, leading to the progression of malignant cancer and poor clinical outcome [14,20]. 

Centromeres are essential to eukaryotic biology by orchestrating the transmission of the genome during cell division. However, although comprising 2% to 5% of the human genome, they are nevertheless still largely a genetic black box [21]. Chromosomal breakpoints at (peri) centromere regions are found in several tumors and are associated with chromosomal instability [22]. Mechanisms leading to centromere breaks are still not well understood [18]. However, pericentromeric instability and breakage are evident in immunodeficiency, centromeric region instability and facial anomalies (ICF) syndrome, caused by defects in DNA methylation due to the impairment of DNA methyltransferase activity [23,24].

Various methods have allowed the identification of several aberrations involving centromeres and telomeres in patients and populations exposed to genotoxic agents. We recently demonstrated that the introduction of telomere and centromere (TC) staining followed by M-FISH (TC+M-FISH) not only renders the analysis of chromosomal aberrations more efficient and robust, but also permits the detection of specific configurations of dicentric chromosomes and their persistence, highlighting the importance of these configurations [25,26].

Here, we demonstrate that combining inverse DAPI and TC+M-FISH allows enhanced detection of chromosomal aberrations and, in particular, of dicentric chromosomes. In addition, telomere length and aberrations can be assessed. We provide validation of this approach in the detection of telomere dysfunction and chromosomal instability. Our technique is readily applicable to the research lab and clinic.

## 2. Materials and Methods 

### 2.1. Materials

Peripheral blood lymphocytes were obtained from a cohort of 50 patients with hematopoietic malignancies and 44 with genetic disorders (Table 1). One hundred healthy donors were used as a control. Cytogenetic preparations were produced from patients and healthy donors. TC+M-FISH was performed to characterize telomere dysfunction and chromosomal aberrations. The collection of blood samples from patients and donors was approved by the Ethics Committee of Gustave Roussy Cancer Campus University Paris Saclay (approval number 97-06). 

### 2.2. Methods

#### 2.2.1. Preparation of Metaphase Spreads

Peripheral blood lymphocytes were cultured in RPMI1640 and exposed to colcemid (0.1 µg/mL) (Gibco KaryoMAX, ThermoFisher Scientific, Les Ulis, France) for 2 h at 37 °C, 5% CO2, in a humidified atmosphere, to arrest dividing cells in metaphase. After harvesting the cells, they were centrifuged for 7 min at 1400 RPM at room temperature, the supernatant removed, and the cell pellet re-suspended in a solution of warm (37 °C) 0.075 M potassium chloride (KCl) (Merck, Kenilworth, NJ, USA) and incubated for 20 min in a 37 °C water bath (hypotonic shock). The cells were pre-fixed by adding approximately five drops of fixative (3:1 ethanol/acetic acid) to each tube under agitation and the tubes centrifuged for 7 min at 1400 RPM at room temperature. The supernatant was removed and the cells suspended in the fixative solution and centrifuged using the same parameters. After two additional rounds of these fixation steps, the cells were stored in the fixative solution at 4 °C overnight and the metaphases spread on cold wet slides the next day. The slides were dried overnight at room temperature and stored at −20 °C until further use. 

#### 2.2.2. TC+M-FISH

Telomeres and centromeres were stained with a Cy-3-labelled PNA probe specific for TTAGGG for telomeres and a FITC-labeled probe specific for centromere sequences (obtained from Eurogentec, Leige, Belgium), as described in M’kacher et al. [25,27]. Briefly, slides were washed with 1X PBS and fixed with 4% formaldehyde at room temperature. After rinsing three times with PBS, the slides were treated with pepsin (0.5 mg/mL) at 37 °C for 5 min. After rinsing three times with PBS, the slides were sequentially dehydrated with 50%, 70%, and 100% ethanol and air-dried. The telomere and centromere probes were added to the slides and subsequently denatured on a hot plate at 80 °C for 3 min and then incubated in the dark for 1 h at room temperature. The slides were subsequently rinsed with 70% formamide/10 mM Tris pH 7.2 three times during 15 min and then in 50 mM Tris pH7.2/150 mM NaCl pH 7.5/0.05% Tween-20 (3 × 5 min). After a final rinse in PBS, the slides were counterstained with DAPI and mounted in PPD at the appropriate pH. After telomere quantification and the automatic capture of metaphases with telomere and centromere staining, the slides were washed with 2X SCC for 30 min at 70 °C. After rinsing with 0.1X SSC, the slides were denatured using NaOH and subsequently washed with 0.1X SCC and 2X SSC and sequentially dehydrated in 70%, 95%, and 100% ethanol and air-dried. After denaturation of the M-FISH probe (MFISH 24XCyte, Metasystems, Altlussheim, Germany) for 5 min at 75 °C, the probe was added to the slides and the slides incubated at 37 °C for two days. The slides were subsequently rinsed with 0.4X SSC for 2 min at 72 °C and then in 2X SSC/0.005% (Tween-20). The slides were counterstained with DAPI and mounted in PPD [25,26].

#### 2.2.3. Telomere Quantification

Two approaches were developed for the quantification of telomere length using TeloScore software. The first consisted of the quantification of telomeres in interphase cells, permitting the investigation of intercellular variation in a large number of scored cells. Quantitative image acquisition was performed using MetaCyte software (MetaSystem, version 3.9.1, Altlussheim, Germany). The exposure and gain settings remained constant between captures. The analysis was performed using TeloScore Software (Cell Environment, Paris France). The mean fluorescence intensity (FI) of telomeres was automatically quantified and analyzed in 10,000 nuclei on each slide. The experiments were performed on triplicate slides. The second approach consisted of quantifying telomere length in metaphases using ChromoScore Software (Cell Environment, Paris France). The images of metaphases were captured using automated acquisition module Autocapt software (MetaSystems, version 3.9.1) and a ZEISS Plan-Apochromat 63×/1.40 oil (Zeiss, Oberkochen, Germany) and CoolCube 1 Digital High-Resolution CCD Camera (MetaSystems, Altlussheim, Germany) with constant settings for exposure and gain. The telomere lengths of individual chromosomes, as well as the mean telomere length of metaphases, were measured. 

Telomere length, measured as the mean FI, strongly correlated with telomere length measured by conventional Southern blot analysis using the telomeric restriction fragment (TRF) (R^2^ = 0.721 and *p* = 2.128 × 10^−8^). The mean telomere length is expressed in kb.

#### 2.2.4. Scoring of Telomeres and Chromosomal Aberrations

For each patient, telomere and chromosomal aberrations were analyzed on an average of 100 metaphases. Telomere aberrations were assessed after telomere and centromere staining: (i) telomere loss was defined as a signal-free end at a single chromatid, an aberration that leads to telomere end fusion and breakage–fusion–bridge cycles [28]; (ii) telomere doublets or telomere fragility were defined as more than one telomere signal at a single arm, an aberration signaling inadequate telomere replication and the dysfunction of shelterin proteins [29,30]; (iii) telomere deletion was defined as the loss of two telomere signals on the same arm, an aberration considered to represent double strand breaks, leading to the activation of DNA repair mechanisms. Automatic scoring of these aberrations was performed using ChromoScore software (Cell Environment, Paris, France), and an operator validated and excluded the false aberrations. Ikaros software (MetaSystems) was used for the classification of chromosomes following conventional cytogenetic G or R-Banding, and Isis software (MetaSystems) was used after M-FISH. In addition to the scoring of translocations, insertions and deletions, TC+M-FISH allowed the scoring of dicentric chromosomes, centric rings, and various types of acentric chromosomes.

#### 2.2.5. Statistical Analysis 

Data were analyzed by Wilcoxon–Mann–Whitney rank sum test or the Kruskal–Wallis non-parametric test. A *p* value < 0.05 was considered statistically significant.

## 3. Results

### 3.1. Improved Identification of Chromosomal Aberrations after TC Staining

**Figure 1:**
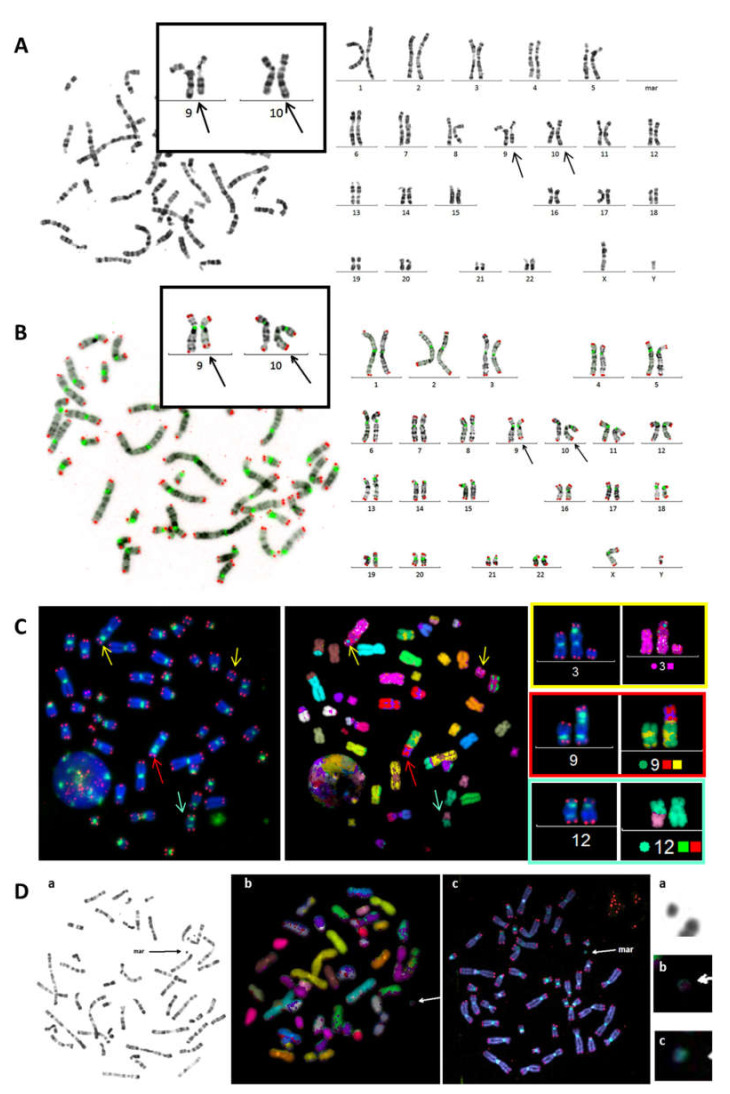
Cytogenetic detection of chromosomal aberrations. (**A**) R-banding, based on the morphological criteria of chromosomes, was the first and widely used technique for clinical cytogenetics. The detection of clonal aberration t (9;10)(q34;q2?6) in a renal-tract malformation patient without information on the precise breakpoint of chromosome 10 or the nature of translocation (balanced or unbalanced). (**B**) Telomere (red) and centromere (green) staining allows reliable classification of chromosomes and identification of chromosomal aberrations, such as t(9;10)(q34;q26.3), with precise localization of breakpoints confirming the reciprocal translocation. This particular reciprocal translocation involved the telomere region of chromosome 10. (**C**) The precise detection of the centromeric regions identifies all chromosomal aberrations, including dicentric chromosomes, in particular when both centromeres are very close, such as a tric (red arrow). The M-FISH technique does not stain the centromeric region. Telomere and Centromere staining followed by M-FISH technique (TC+M-FISH ) permits the reliable scoring and identification of all chromosomal aberrations, such as the dic (12;17), which could be mistaken for two chromosomes using only M-FISH. (**D**) Detection of a centric ring in circulating lymphocytes of a patient with a genetic syndrome using TC staining (**c**). This centric ring was undetectable by R-Banding(**a**) or M-FISH (**b**).

Identification of chromosomal aberrations using conventional cytogenetic techniques is based on chromosome morphology and G and/or R-Banding or inverted DAPI staining. These approaches allow the identification of chromosomes according to their size and specific banding patterns (Figure 1A). The introduction of centromere staining allowed visualization of the centromeric regions and, as a result, the identification of dicentric chromosomes, centric rings, and acentric fragments. Furthermore, telomere staining made it possible to identify chromosome ends and improved the identification of chromosome territories, particularly in the case of overlapping chromosomes, and thus the detection of chromosomal aberrations. TC staining also made it possible to eliminate false-positive and false-negative aberrations, which are impossible to detect with conventional cytogenetics. In addition, TC staining improved classification of chromosomes related not only to their size, but also to the size of the p and q arms. Moreover, inverted DAPI, similar to GTG banding, improved the efficiency of the identification of chromosomes and the detection of their aberrations (Figure 1B). TC staining also improved the detection of dicentric chromosomes with centromeres in close proximity to or in contact with the telomeres (Figure 1C). It is now possible to score and distinguish very small centric and acentric rings more accurately. Hitherto, this configuration has been extremely difficult to detect using conventional and molecular approaches (Figure 1C,D). 

**Figure 2:**
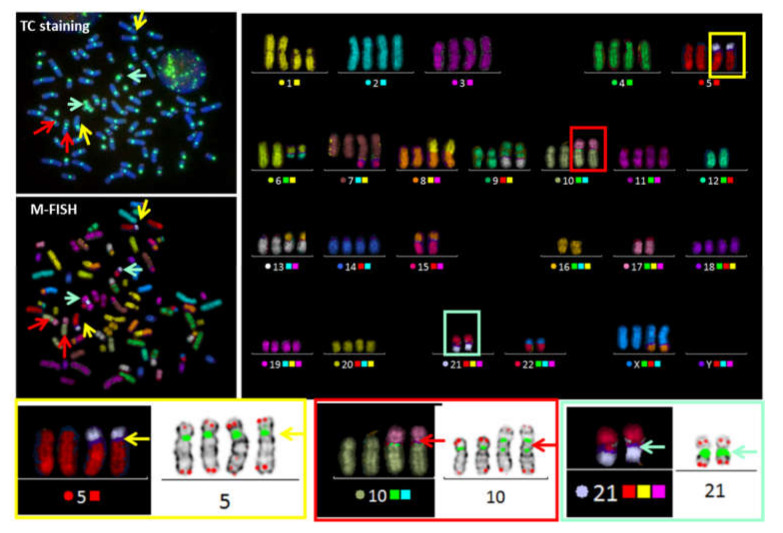
TC+M-FISH used to detect a complex karyotype in a case of acute lymphoblastic leukemia. This approach makes it possible to detect not only the translocation and complex exchange, but also the presence of a specific configuration of dicentric chromosomes with both the centromeres juxtaposed, such as dic(5;21)(p10;p10) and dic(21;22)(p10;p10) (arrow yellow and cyanine), or when the distance between both centromeres is very small (dic(10;17)(p10; p11).

In cases of a complex karyotype, TC+M-FISH allows enhanced visualization of repeated sequences (telomeres and centromeres), which are undetectable using M-FISH on its own, and is also a more reliable method for the detection of chromosomal aberrations. Dicentric chromosomes with both centromeres in close proximity may be mistaken for translocations using M-FISH alone. The detection of dicentric chromosomes with a specific configuration can be achieved using TC staining followed by M-FISH technique. The power of this approach to identify characteristic dicentric chromosomes in the blood cells of an acute lymphoblastic leukemia patient with both centromeres in close contact is demonstrated in Figure 2.

### 3.2. Telomere Instability Detected by TC+M-FISH Staining

Telomere instability is defined by telomere shortening and/or telomere dysfunction (uncapped or damaged telomeres) and is considered to be an important mechanism underlying chromosomal instability. Thus, telomere instability may be a key player in the process of oncogenesis. However, the absence of a proper technique to detect telomere instability adapted to the clinical routine has hitherto led to global underestimation of its role. The introduction of TC staining in cytogenetics for clinical investigation now permits the assessment of telomere length and instability. 

After TC staining, the quantification of telomere length can be performed in interphase nuclei, as well as in metaphases. 

**Figure 3:**
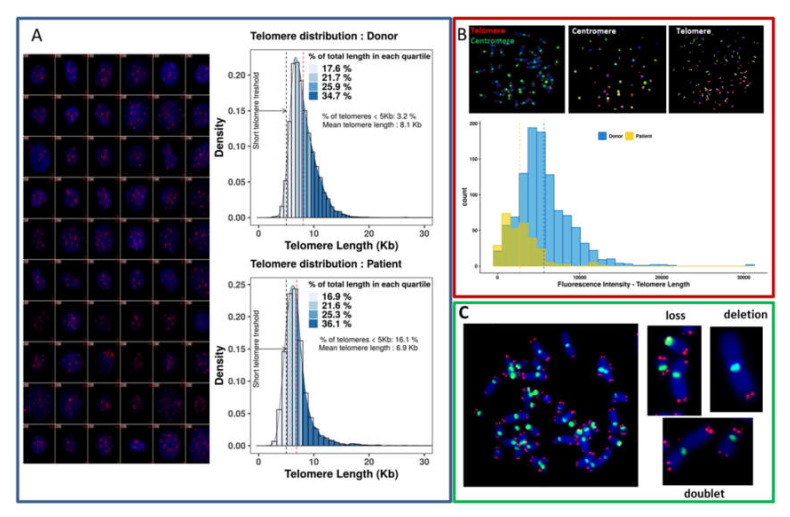
Quantification of telomere length and telomere dysfunction. (**A**) Global quantification of telomere lengths in nuclei allows their assessment in a large number of cells and analysis of the intercellular heterogeneity of telomere lengths. An example of global quantification of telomere length in a healthy donor and a cancer patient is shown using TeloScore software. Our technique permits analysis of the mean telomere length, the distribution of fluorescence intensity in each quartile and the frequency of cells with telomeres < 5 kb. The distribution demonstrates the heterogeneity of telomere length. A significant difference between cells from the healthy donor and the cancer patient in the frequency of cells with drastic telomere shortening (< 5 kb) (black line) is demonstrated. However, mean telomere length (red line) is not always an appropriate indicator. (**B**) The use of cytogenetic slides for quantification of telomere length offers the possibility to detect telomere sequences in individual chromosomes, quantification of telomeres, and assessment of intrachromosomal variations of telomere length. Significant differences are observed in the intensity of each telomere in metaphases from a healthy donor and a cancer patient, the heterogeneity being greater for the cancer patient (**C**) The use of metaphases permits detection of telomere aberrations such as telomere loss, telomere deletion (the loss of two telomeres in the same arm), and the formation of telomere doublets. All these telomere aberrations are related to telomere dysfunction.

Global quantification of the fluorescence intensity of the telomeric sequences in nuclei permits the detection of not only the mean telomere length, but also the inter-cellular variation and proportion of cells with drastic telomere shortening in a large number of nuclei (Figure 3A).

The quantification of telomere length can be performed on metaphases, allowing measurement of the intensity of the fluorescence of each telomere signal. This approach allows analysis of the intra-chromosomal variation and heterogeneity of telomere signals in metaphases (Figure 3B).

TC staining is a unique technique that permits the analysis of telomere loss, telomere deletion, and the formation of telomere doublets for each chromosome, offering the possibility to assess telomere aberrations after the classification of chromosomes has been performed in metaphases (Figure 3C).

### 3.3. Validation of the Concept 

#### 3.3.1. Telomere Dysfunction

**Figure 4:**
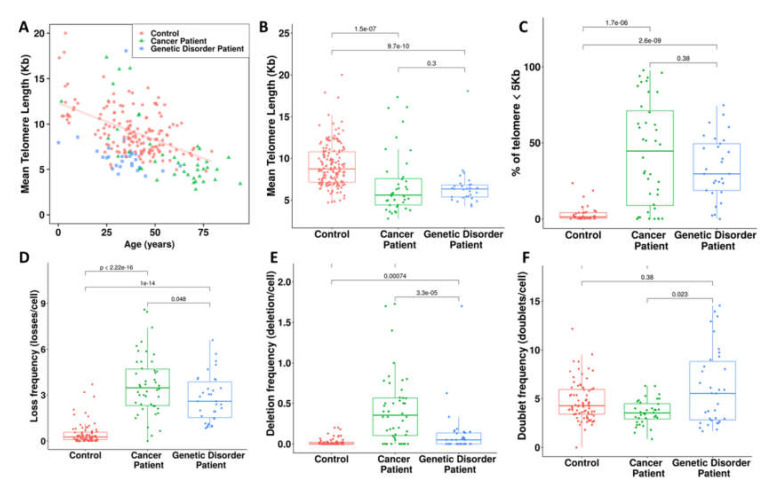
Telomere dysfunction in cancer patients and those with genetic syndromes. (**A**) Telomere length of healthy donors is age-dependent. The regression line indicates telomere shortening with age in healthy donors (79 pb per year; Y = 12.1−0.79X; R^2^ = 0.29). In cancer patients and those with genetic disorders, there is no significant correlation between telomere length and age. High individual variation is observed in telomere length of healthy donors, cancer patients, and genetic disorder patients. (**B**) Cancer patients and those with genetic syndromes show significantly shorter telomeres than healthy donors. (**C**) Analysis of the frequency of cells with short telomeres (<5 kb) reveals a significant difference between cancer patients and those with genetic disorders and healthy donors. (**D**) The frequency of telomere loss, the major telomere aberration that leads to telomere fusion and dicentric chromosome formation, is significantly higher in cancer patients and those with genetic syndromes than in healthy donors. (**E**) Similarly, the frequency of telomere deletions is significantly higher in cancer patients and those with genetic disorders than in healthy donors. (**F**) There is no significant difference in telomere doublet formation between healthy donors and patients with genetic disorders.

Quantification of telomere lengths using this approach demonstrated that telomere lengths in healthy donors are related to age, with high inter-individual variation. In healthy donors, the mean decrease of telomere length was 79 bp/year (Figure 4A). Patients with cancer or genetic disorders displayed a significant reduction in mean telomere length relative to controls (*p* < 10^−7^ for cancer patients; *p* < 10^−10^ for patients with genetic disorders) (Figure 4B). This difference in telomere length between cancer patients and those with genetic disorders and controls increased when we instead analyzed the frequency of cells with major telomere shortening (<5 kb) (*p* < 10^−13^ for cancer patients and those with genetic disorders) (Figure 4C). 

**Figure 5:**
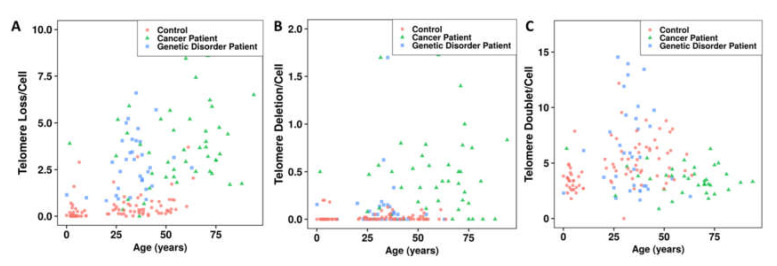
Variation of telomere dysfunction with age in healthy donors, cancer patients, and patients with genetic disorders: telomere dysfunction is relatively independent of age in all groups: (**A**) telomere loss, (**B**) telomere deletion, and (**C**) telomere doublet formation.

The analysis of telomere loss, deletion, and doublet formation in healthy donors demonstrated that these telomere aberrations are age-independent (Figure 5). In cancer patients and those with genetic disorders, we observed higher frequencies of telomere loss (*p* < 10^−13^ and *p* < 10^−13^, respectively) (Figure 4D), telomere deletions (*p* < 10^−8^; *p* < 10^−2^, respectively) (Figure 4E), and age independence (Figure 5A,B) than in healthy donors. However, the frequencies of telomere loss and deletions in cancer patients (mean age 45 years) were significantly higher than those observed in genetic disorders patients (mean age 32 years) (*p* = 0.048 and *p* = 3.3 × 10^−5^, respectively). 

Of note, significantly less telomere doublet formation was detected in cancer patients than in healthy controls and patients with genetic disorders (*p* = 0.01 and *p* = 0.02, respectively) (Figure 4F and Figure 5C). Moreover, there was no significant difference between the frequency of telomere doublet formation in patients with genetic syndromes and healthy donors (Figure 4F and Figure 5C). 

### 3.3.2. Detection of Dicentric Chromosomes

**Figure 6:**
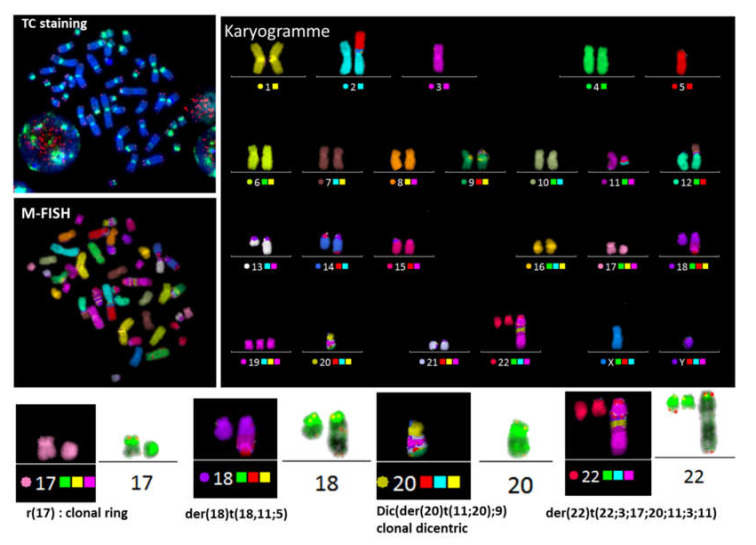
The presence of dicentric chromosome is associated with telomere dysfunction and complex karyotypes. Sequential analysis shows the presence of clonal dicentric chromosomes and centric rings in a mantle-cell lymphoma patient. These configurations are related to the presence of chromosomal aberrations related to breakage–fusion–bridge cycles, such as der(18) t (18,11;5) with an interstitial telomere of chromosome 18 and der(22) t (22;3;17;11;3;11).

Dicentric chromosomes are considered to be an important biomarker of chromosomal instability and are associated with complex karyotypes and poor clinical outcome [31]. Therefore, the identification of dicentric chromosomes in patients with cancer or genetic disorders may facilitate identification of the disease and thus support the decision for an optimal therapeutic strategy. Dicentric chromosomes were found in 28/50 hematological cancer patients and 20/44 patients with genetic disorders using TC+M-FISH vs. 7/50 and 12/44 by conventional cytogenetics, respectively. A specific dicentric chromosome configuration, in which the two centromeres are close to each other, was found in 70% of cases of cancer patients and those with genetic disorders. Such dicentric chromosomes are easily missed by conventional cytogenetics as well as molecular approaches and can be mistaken for translocations (Figure 3). In addition, the detection of dicentric chromosomes was related to the presence of a complex karyotype in cancer patients, as well as those with genetic disorders (Figure 6).

**Figure 7:**
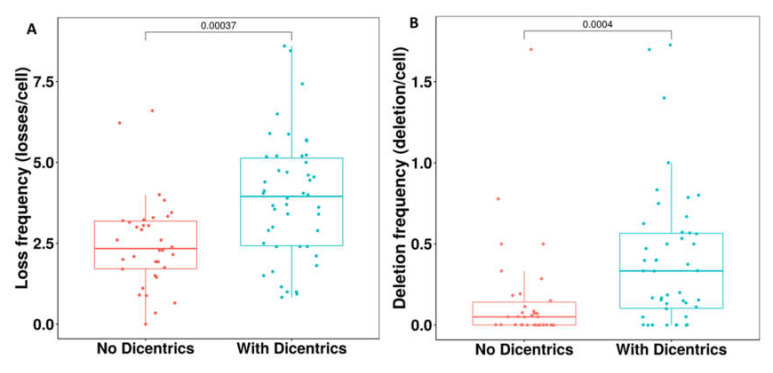
Telomere dysfunction and dicentric chromosome formation. (**A**) High frequency of telomere loss and (**B**) deletion in patients with dicentric chromosomes compared to those without.

There was a significant correlation between the presence of dicentric chromosomes and telomere loss (*p* < 10^−4^) (Figure 7A) and telomere deletion (*p* < 10^−4^) (Figure 7B). This underscores the importance of impaired telomere integrity in the initiation and progression of chromosomal instability. In contrast, no significant correlation was observed between telomere length and the presence of dicentric chromosomes. 

### 3.4. Proof of Concept in the Clinics

**Figure 8:**
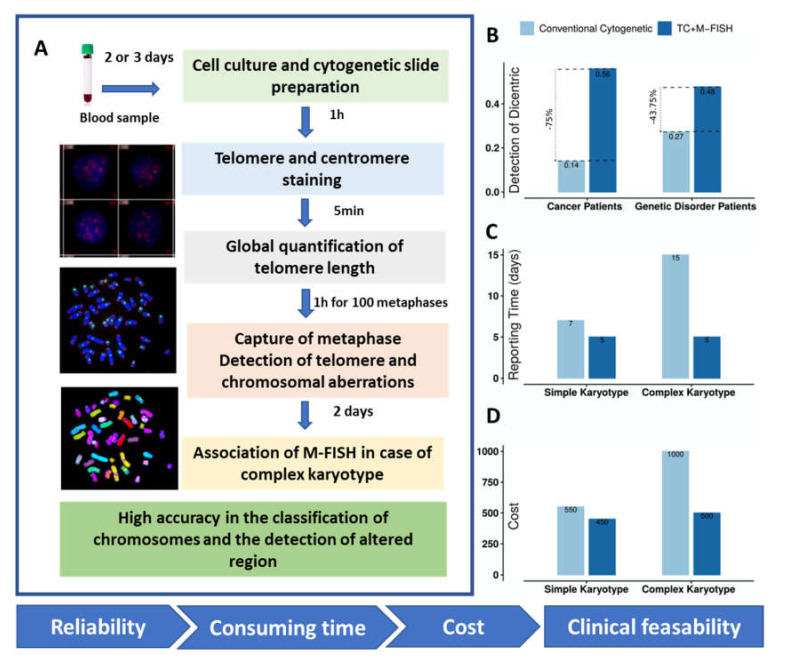
Overview of TC+M-FISH for the detection of chromosomal aberrations (**A**) Description of the TC+M-FISH approach. (**B**) Sensitivity of TC+M-FISH in the detection of dicentric chromosomes in cancer patients and those with genetic disorders compared to conventional cytogenetics. (**C**) Reporting time from the analysis of blood samples from patients, with or without complex karyotypes, using the TC+M-FISH approach compared to conventional cytogenetics (**D**) The cost (in euros) of the two approaches for the analysis of a simple and complex karyotype, based on the European situation.

Reproducibility, feasibility, and low cost are critical for any new technique considered for routine clinical use. We provide data on the evaluation of the time, concordance of results, and cost of testing cancer patients and those with genetic disorders using conventional cytogenetics and the TC+M-FISH approach for chromosomal analysis (Figure 8).

The use of PNA probes allows shorter hybridization times and provides higher signal intensity and lower costs than DNA probes for TC staining. Consequently, the introduction of TC staining among the techniques commonly used in clinical practice should not increase the costs relative to those of conventional cytogenetic analysis. Furthermore, TC staining will dramatically shorten the time devoted to the analysis of the results. In addition, TC-M-FISH is a reliable and sensitive approach for the analysis of a large number of cells and the detection of clonal expansion and chromosomal instability (Figure 8B–D).

The assessment of chromosomal aberrations by TC+M-FISH indeed incurs certain additional costs required for consumables. However, the technique offers higher specificity and reliability of the results, as well as a significant reduction in the time required for analysis relative to that of conventional cytogenetics in cases of simple or complex karyotypes. Moreover, quantification of telomere length and telomere aberrations is not possible by conventional cytogenetics. The application of an automated approach (TeloScore and ChromoScore) renders the global quantification of telomere length, as well as the scoring of telomere and chromosomal aberrations, easier and more reliable.

## 4. Discussion

Chromosomal instability is known to interfere with treatment responses in cancer patients and more generally with clinical outcomes in populations exposed to genotoxic agents [32]. However, until now, the analysis of chromosomal instability has not been incorporated into clinical practice [33]. The lack of standardization and the uncertainties in terms of optimal cut-offs may account for its suboptimal utilization and the consequent absence of proof of its clinical usefulness. 

In particular, the analyses of telomere and centromere sequences have been excluded from cytogenetic studies in clinical practice. However, telomere and centromere staining has greatly contributed to improving our knowledge of their role in carcinogenesis, tumor progression, and genetic disorders. 

Here, we describe an adaptation of a chromosome banding and fluorescence in situ hybridization approach to assess telomere dysfunction and chromosomal instability in cytogenetic clinical practice, not only to identify cancer patients with a high degree of chromosomal instability and improve their therapy, but also to assess the level of cancer risk in patients with a genetic disorder or in a population exposed to genotoxic agents. This allowed the detection of aberrations involving unique and repeated sequences. 

First, we examined telomeres and sub-telomere regions that play a key role in genome stability and integrity [34]. Telomere length is a biomarker of cancers and aging disease [20,35]. 

Although long telomeres may be associated with higher telomerase activity in certain cancers [20,36,37], telomere shortening represents the main mechanism of senescence and tumor initiation and progression. In addition, growing interest in the implication of telomere dysfunction in genetic disorders has been recently addressed [38,39]. The development of a faster and more efficient diagnostic approach for the detection of telomere dysfunction in cytogenetic clinical practice is necessary to not only better target cancer cells and genetic disorders but also to monitor populations exposed to genotoxic agents. Using cytogenetic slides, we demonstrate that our approach, based on the Q-FISH technique, can easily assess mean telomere length and the inter-cellular and the inter-chromosome heterogeneity of telomere signals. In addition, it was possible to assess telomere loss, telomere deletion, and the formation of telomere doublets, which are the consequence of telomere dysfunction. This approach provides multiple advantages for the quantification of telomere length and telomere aberrations. Visualization of telomere signals in each cell and on metaphase chromosomes allows detection of those cells that present very short telomeres and chromosome uncapping, which play a major role in senescence and initiation of diseases [40]. In contrast to most existing high-throughput techniques for telomere quantification, such as quantitative PCR (qPCR) [41] or flow-FISH [42], the heterogeneity of telomere length and telomere aberrations can be scored at the single-cell level and detected on the chromosomes using our approach. Indeed, we show that the mean telomere length from a defined sample is not necessarily the best biomarker for telomere dysfunction. Our data contribute to the validation of the concept that the proportion of cells with short telomeres is a better biomarker for aging and diseases [43]. Surprisingly, telomere loss and deletion, which are age-independent in healthy populations, may be more relevant than mean telomere length or the frequency of cells with short telomeres. Their frequencies were more pronounced in cancer patients and those with genetic disorders. Therefore, these telomere aberrations should be used as more accurate biomarkers for disease risk stratification. Further studies in a large cohort of healthy donors and cancer and genetic disorder patients would be needed to establish the cut-off of these aberrations and confounding factors that influence their frequency.

Next, we analyzed the critical role of centromeres in maintaining genome integrity. Increased evidence has accumulated that centromeric and pericentromeric regions display heterogeneous alterations in several diseases [22]. Conventional and genomic approaches have demonstrated their limited capability to assess aberrations related to these regions [18]. The implication of centromeric and pericentromeric regions in chromosomal aberrations and chromosomal instability is still unresolved. We demonstrated previously that the application of TC+M-FISH improved the detection of chromosomal aberrations and their persistence in subsequent cell generations after exposure to genotoxic agents, such as irradiation [26]. In addition, we demonstrated that the transmission of dicentric chromosomes was more efficient when both centromeres were very close and centromeres were near telomere sequences. Furthermore, small centric rings demonstrated a higher rate of stability. These configurations cannot be detected by conventional staining or molecular cytogenetic approaches. In light of these data, it is necessary to re-evaluate the presence of dicentric chromosomes and centric rings in cancer patients and those with genetic disorders by TC+ M-FISH. The presence of dicentric chromosomes in cancer patients and those with genetic disorders has been related to the presence of chromosomal instability, a complex karyotype, and poor clinical outcomes [44,45,46]. In a cohort of 50 cancer patients and 44 patients with genetic disorders, we re-evaluated the presence of dicentric chromosomes, centric rings, and other chromosomal aberrations to establish a reliable and robust karyotype and detect the heterogeneity or the mosaics that are known to participate in the early steps of tumorigenesis and the initiation of chromosomal instability. Most dicentric chromosomes detected were characterized by a specific configuration, consisting of two centromeres in very close proximity in both populations of patients. These dicentric chromosomes were not detected using conventional cytogenetic approaches (chromosome banding or FISH). These results validate our previous data concerning the transmission of chromosomal aberrations and highlight the implication of the instability of centromeric or peri-centromeric regions in the formation of a specific configuration of dicentric chromosomes [26]. Application of the centromeric FISH technique has previously been reported for the identification of dicentric chromosomes [47,48,49,50,51]. Multi-centromeric FISH has been proposed as a reliable and robust routine technique for the detection of dicentric chromosomes [51]. Dicentric chromosomes were detected in 51% of analyzed patients with acute myeloid leukemia. It was reported that these dicentric chromosomes were characterized by their short intercentromeric distance [48]. Our study confirms these data and provides an attractive approach to cytogenetic clinical practice with a short time of hybridization, robust signals, and a low cost relative to multi-centromeric FISH probes. 

We detected a relationship between telomere dysfunction (telomere loss and deletion) and the presence of dicentric chromosomes in cancer patients and those with genetic disorders. These telomere aberrations appear to be shared between genetic disorders and cancer, with a higher frequency in cancer patients, possibly related not only to the age of these patients but also to the genetic detriment of cancer [52]. Nevertheless, patients with a genetic disorder exhibit a higher risk of developing cancer [53,54]. In addition, we confirm the prominent role of telomere dysfunction in the formation of the configurations of dicentric chromosomes and chromosomal instability. In future, it will be important to elucidate the relationship between telomere dysfunction and the instability of centromeric or pericentromeric regions.

Current genomics techniques have demonstrated their limitations in the detection of chromosomal aberrations involving repetitive sequences, such as telomeres and centromeres. Implementation of our approach as an adjunct to the detection of chromosomal instability makes it possible to improve the automatic detection of chromosomal aberrations at a high sample throughput for routine clinic processes and follow-up of populations exposed to genotoxic agents. Chromosome banding and FISH of telomeres and centromeres provide more accurate and time-saving detection of these chromosomal aberrations. This technique also paves the way for more efficient guided genomic studies, including NGS investigations. In addition, these data represent a first step in the establishment of the bridge between the genome and the chromosome [55], permitting better detection of chromosomal aberrations, and the introduction of this approach to the clinic, providing higher efficiency at a low cost. These data could help define potential therapeutic strategies including telomeres [56,57].

## 5. Conclusions

This study demonstrates the potency of the TC+M-FISH technique as a reliable and robust method for the detection of telomere dysfunction and chromosomal aberrations. Our data encourage implementation of this technique as a routine method for research as well as clinical uses. We suggest that automation of the entire process, determination of the background of chromosomal aberrations and telomere dysfunction in the general population, and improvement of databases will make it possible to improve detection of chromosomal aberrations and chromosomal instability in the clinic. 

## 6. Patents

The patented process number is FR1858427 and WO2020058268.

## Figures and Tables

**Table 1:** Characteristics of Cancer patients and genetic disorders patients.

Characteristics	No. of Patients
**Cancer patients**	50
Male	32
Age (years)	56
Type	
Hodgkin lymphoma	18
Non Hodgkin lymphoma	15
Mantel Cell lymphoma	8
B-cell prolymphocytic leukemia	2
Myelodysplasia syndrome	4
Other	5
**Genetic syndrome**	44
Male	27
Age (years)	32
Type	
Turner syndrome	9
Down syndrome	4
Klinfelter syndrome	2
Li-Fraumeni	2
Telomerepathies	2
Other *	15

* rare genetic diseases and malformative syndromes.
